# Early orthoplastic coverage reduces fracture-related infections in Gustilo III B/C tibial fractures – a systematic review and meta-analysis

**DOI:** 10.1007/s00068-026-03189-2

**Published:** 2026-04-24

**Authors:** Neil Ruprecht, Felix Karl-Ludwig Klingebiel, Christian Thomas Hübner, Jakob Hax, Henrik Teuber, Kai Oliver Jensen, Christian Hierholzer, Roman Pfeifer, Hans-Christoph Pape, Valentin Neuhaus, Yannik Kalbas

**Affiliations:** 1 Harald-Tscherne Laboratory for Orthopaedic and Trauma Research, University Hospital Zurich, University of Zurich, Rämistrasse 100, Zurich, 8091 Switzerland; 2 Department of Trauma Surgery, University Hospital Zurich and Harald-Tscherne Laboratory University of Zurich, Rämistrasse 100, Zurich, 8091 Switzerland; 3 Department of Hip and Knee Surgery, Schulthess Clinic, Lengghalde 2, Zurich, 8008 Switzerland

**Keywords:** Open tibial fractures, Gustilo-anderson Type III B/C, Orthoplastic coverage, Soft tissue coverage, Fix and flap, Fracture-related infection, Meta-analysis

## Abstract

**Background:**

Gustilo-Anderson type III B/C open tibial fractures are among the most complex and high-risk injuries in trauma care. Their management demands a multidisciplinary approach, and early intervention is critical. While early debridement has been established as essential, the optimal timing for definitive orthoplastic coverage remains less clearly defined.

**Objective:**

To evaluate whether early orthoplastic coverage reduces the risk of fracture-related infections (FRIs) and complication rates compared to late coverage in type III B/C open tibial fractures.

**Methods:**

We conducted a PRISMA-compliant systematic review and meta-analysis of studies comparing early versus late orthoplastic treatment in patients with Gustilo-Anderson III B/C open tibial fractures. Databases included PubMed and EMBASE. Primary outcome was FRI; secondary outcomes included amputation, non-union, and revision surgery rates. Risk of bias was assessed using the Newcastle-Ottawa Scale.

**Results:**

Of 3746 studies screened, five studies with a total of 454 patients met the inclusion criteria. Cutoff for early vs. late treatment was defined variably across studies (either within 72–168 h). Meta-analysis showed that early treatment significantly reduced the odds of infection (OR: 0.34; 95% CI: 0.23–0.52; *p* < 0.001) compared to late treatment. Subgroup analyses confirmed consistent benefits regardless of timing cutoffs or surgical approach (“fix and flap” vs. staged). While trends suggested reduced rates of amputation and non-union with early treatment, these did not reach statistical significance.

**Conclusion:**

Early orthoplastic coverage significantly reduces FRI in patients with Gustilo-Anderson III B/C open tibial fractures. While secondary outcomes showed positive trends, further high-quality prospective studies are needed.

**Supplementary Information:**

The online version contains supplementary material available at https://doi.org/10.1007/s00068-026-03189-2.

## Highlights

This meta-analysis investigates the timing of orthoplastic treatment in Gustilo-Anderson III B/C open tibial fractures.

Early orthoplastic coverage significantly reduces the risk of fracture-related infections compared to delayed treatment.

The benefits of early treatment remained consistent regardless of timing cut-offs (72 vs. 168 h) or surgical approach (staged vs. “fix and flap”).

Secondary outcomes such as amputation and non-union trended favorably with early treatment but lacked statistical significance.

**Supplementary Information:**

The online version contains supplementary material available at https://doi.org/10.1007/s00068-026-03189-2.

## Introduction

Gustilo-Anderson Type III B/C open tibial fractures represent a significant portion of the severe open fractures, which are often associated with high energy trauma, frequently lead to infection, and negatively affect fracture healing [1–3]. These fractures are challenging to treat and are often associated with high morbidity and significant socio-economic costs [4, 5]. Due to the extensive soft tissue damage these fractures are best treated via a multidisciplinary approach and should be transferred to a specialist center to receive orthoplastic care [6, 7].

While many studies have already shown that early (< 24 h) and thorough debridement is key to reducing complication and infection rates in severe open fractures [8–10], timing of orthoplastic coverage is less well researched. Few studies suggest that earlier definitive orthoplastic coverage is superior to late orthoplastic coverage [1, 11–14]. However, the literature confirming this is sparse. Therefore, the aim of this study was to identify clinical studies that investigated the effect of early versus late orthoplastic coverage on morbidity in patients with Type III B/C open tibial fractures and to perform a quantitative synthesis of the available evidence.

Our research questions were the following:

1. What are the effects of early versus late orthoplastic treatment on the rate of fracture-related infections (FRI) in patients with Type III B/C open tibial fractures?
2. What are the effects of early versus late orthoplastic treatment on other relevant outcomes like the amputation rate, revision and non-union rate?
3. What is the quality of the available evidence and the agreement within the literature regarding this topic?

## Methods

The reporting of this meta-analysis is in accordance with the Preferred Reporting Items for Systematic Reviews and Meta-Analyses (PRISMA 2020) guidelines [15]. We performed a systematic search to identify all relevant original publications that investigated the effect of early vs. late orthoplastic surgery for fractures of the tibia classified as Gustilo-Anderson III B/C fractures.

### Search strategy

The search was conducted in November, 2025 in the Pubmed and MEDLINE databases: We used a combination of controlled vocabulary and regular search terms. In the Pubmed Database, we included the Medical Subject Headings (MeSH) “Fractures, Open”, “Tibial fracture”, “tibia and open fracture”, “IIIB and open fracture”, “IIIB and Gustilo” in combination with the terms “orthoplastic”, “Fix and Flap”, “myocutaneous flap”, “muscle flap”, “fasciocutaneous flap” or “timing and coverage”. In the EMBASE database, the same terms were searched. Truncation was used to account for plural forms and alternate spellings. Terms were connected by the Boolean operators and filters were applied to exclude inappropriate article types. A comprehensive overview of the search terms is available in the supplements. In addition to the database search, experts in the field of trauma surgery were asked for potentially relevant studies and references lists of selected studies and related reviews were screened, to identify any important studies missed by the electronic search (Additional sources).

### Extraction and screening

Search results were extracted and documented using Rayyan.ai [16], a platform that helps to conduct systematic reviews. Articles were de-duplicated and screened independently by two authors. A cross-check of the extracted data was performed by the last author. Any disagreement was resolved by a consensus discussion in personal meetings.

### Inclusion criteria

Original studies reported in English or German were assessed for inclusion. Articles were included if they investigated Patients with Gustilo Anderson III B/C fractures of the tibia and if they compared an early vs. late orthoplastic approach. While we did not define a cutoff between early and late treatment, it was required for inclusion that the study defined an exact time as cutoff between the early and the late treatment group. Furthermore, studies had to compare the rates of FRI in those two respective groups.

### Exclusion criteria

Letters, commentaries, correspondences, conference abstracts, expert opinions, editorials and in vitro/animal experiments were excluded. Further exclusion criteria were studies that did not use the Gustilo Anderson Classification or studies that used the Gustilo Anderson Classification but did not investigate tibial fractures. We also excluded articles that did not investigate infection rates as well as studies that did not stratify groups for early and late orthoplastic approach. To facilitate a meta-analysis of the results, we had to exclude studies that compared infection rates in relation to the time of debridement without providing numbers for specific timeframes. Furthermore, we excluded studies with a high risk of bias.

### Definitions and outcomes

#### Type III B/C open fractures

Open fractures were classified according to Gustilo-Anderson. The Gustilo-Anderson classification was established in 1976 and constitutes the most commonly used classification for open fractures [4]. It classifies open fractures based on wound size, level of contamination and the extent of soft tissue/osseous injury [4, 17, 18] (see Table 1). Gustilo Type I to III A are less severe and do not regularly require soft tissue coverage whereas Gustilo Grade IIIB and IIIC typically require complex reconstructions and flap coverage and are more prone to complications, especially FRI [19].

**Table 1:** Recapitulation of the gustilo-anderson classification

Gustilo-Anderson Classification:	Description
Type I	Clean open fracture with less than 1 cm wound
Type II	1–10 cm laceration, with moderate soft tissue damage
Type III A	More than 10 cm wound with adequate soft-tissue coverage despite extensive laceration
Type III B	Severe form and is associated with a significant soft tissue loss of more than 10 cm that needs cover
Type III C	Open fracture associated with arterial injury requiring repair

#### Early versus late orthoplastic coverage and surgical strategy

The definition of what constitutes an ‘early’ or a ‘late’ definitive treatment was made by the authors of the included studies and therefore the cutoffs are not consistent. Some studies defined ‘early’ as treatment within 72 h, while others used a cutoff of 168 h. Both cutoffs, 72 h and 168 h were included in the meta-analysis. Additionally, we performed subgroup analyses within the different cut-offs. Furthermore, there were differences in the surgical strategies used between the studies. While in some studies, definitive treatment was performed with a single combined orthoplastic approach (“fix and flap”), others used a staged procedure. We also performed subgroup analysis for each surgical strategy used.

#### Primary outcome

Our primary outcome was the prevalence of FRI in the early versus the late treatment group. Fracture-related infections were defined according to the criteria outlined by Metsemakers et al. in 2018 [20].

#### Secondary outcomes

Our secondary outcomes were the rates of amputation, non/delayed unions and revision rates in the early versus the late treatment group. Non-union was defined as a fracture that has not healed after 9 months, based on the widely accepted criteria established by the FDA in 1998 [21]. Revision rates included any surgical procedure performed after the initial operation to address complications such as hardware removal or replacement due to infection or mechanical failure, as well as bone grafting for non-union and revisions related to flap failure. Amputations were not classified as revisions.

### Retrieval and processing

After initial screening and selection were completed, articles were retrieved from the respective publishers through the access of our universities’ central library. Articles were retrieved and stored as PDFs and organized using EndNote™ (version 20, Clarivate™). Data was collected manually and transferred into MS Excel. Besides general metadata, articles were scanned for number of patients, cutoff times between early and late orthoplastic coverage, the application of single stage orthoplastic coverage (“fix and flap”) and the primary and secondary outcomes (FRI, number of revisions, amputations and non/delayed union rates).

### Eligibility risk of bias assessment

Articles were checked for eligibility by two authors and, in case of uncertainty, cross-checked with the last author in personal meetings. Risk of bias was evaluated with the Newcastle-Ottawa Scale by the first and last author and graded as “low”, “moderate” and “high” [22]. Any disagreement was resolved in a personal meeting. Studies with “low” and “moderate” risk of bias were included in the synthesis. An overview of the Risk of Bias assessment is presented in the supplemental material (Table S1).

### Data analysis

Continuous, normally distributed data were reported as means with standard deviations (SD), while non-normally distributed continuous or ordinal data were presented as medians with interquartile ranges (IQR). Categorical variables were summarized using frequencies and percentages. Statistical analysis was performed using R [R Core Team, 2024]. Meta-analyses were conducted using the inverse-variance method under a common effects model. The effect size was expressed as an odds ratio (OR) with a corresponding 95% confidence interval (CI). The primary comparison evaluated the incidence of FRI following early versus late orthoplastic intervention. All meta-analytic procedures were conducted using the **meta** package in R [23]. Statistical significance was defined as a p-value < 0.05. Heterogeneity among studies was assessed using Cochran’s Q test and quantified with the I² statistic, where values of 25%, 50%, and 75% were considered low, moderate, and high heterogeneity, respectively. Tau² was also calculated to estimate between-study variance. An I² of 50% was chosen as the cutoff to report results from the random effects model instead of the fixed one. Due to the small number of included studies, formal statistical tests for publication bias (e.g., Egger’s test) were not performed. However, visual inspection of the funnel plots did not indicate major asymmetry.

## Results

### Study selection

**Fig. 1:**
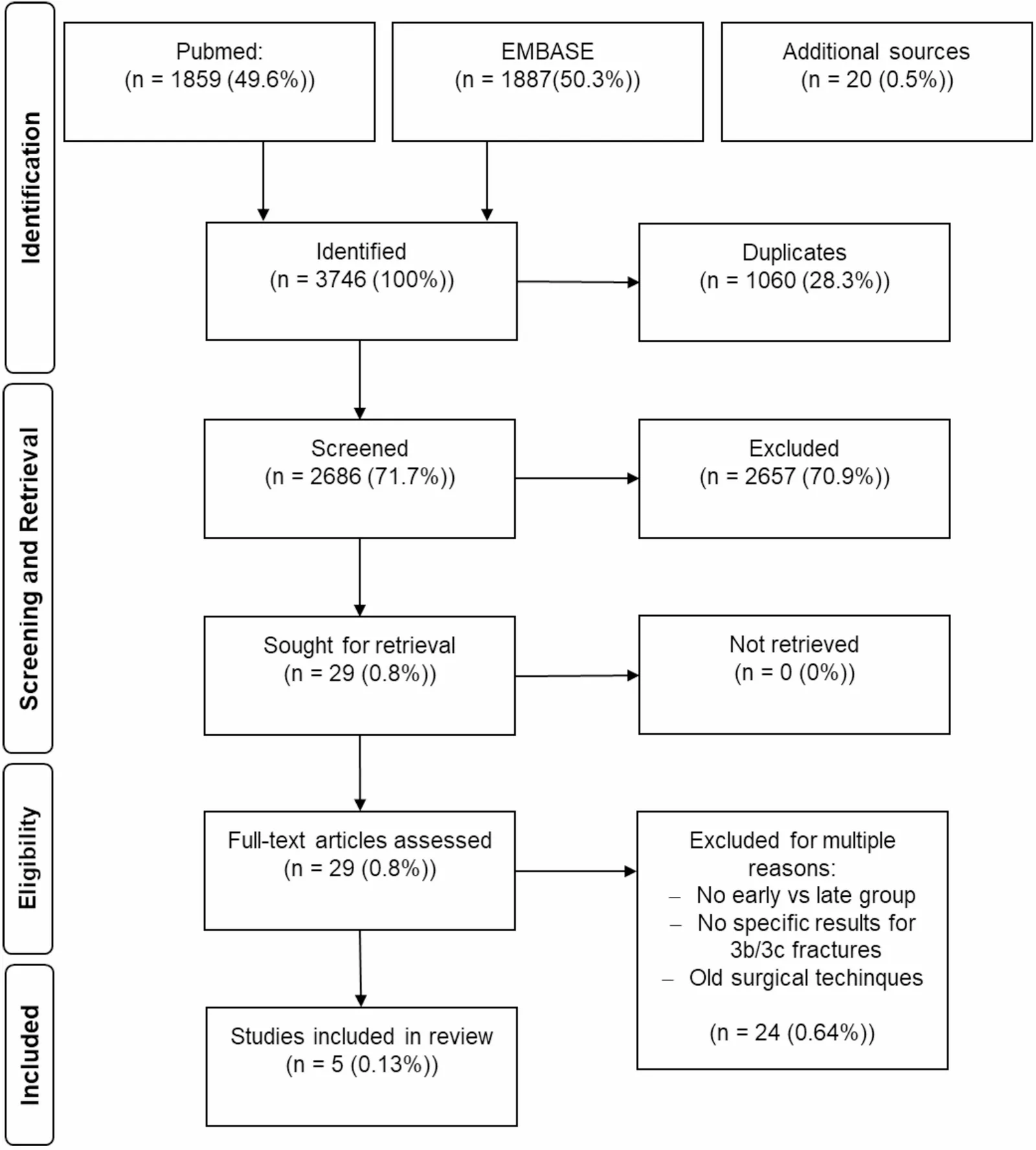
PRISMA Flowchart of the study selection process

The PRISMA-flowchart of the study selection process is presented in Fig. 1. The systematic search in the Embase Database yielded 1887 results and 1859 records were identified through Pubmed. A further 20 records were identified through additional sources. After removing 1060 duplicates, 2686 records were screened, with 2657 excluded during initial screening. The remaining 29 articles were sought for retrieval. All 29 full-text articles could be retrieved and were assessed for eligibility. After exclusion of 24 articles, 5 studies remained for quantitative synthesis [24–28]. (An overview of article metadata is presented in Table 2).

**Table 2:** Synopsis of metadata, number of participants, cutoff between early and late orthoplastic coverage, utilization of a single-stage “Fix and Flap” approach

Author / Year	Title	Journal	Region	*N* =	Cutoff Early vs. Late	“Fix & Flap”
Aljawadi et al. 2020	One-stage combined ‘fix and flap’ approach for complex open Gustilo-Anderson IIIB lower limbs fractures: a prospective review of 102 cases	Archives of Orthopaedic and Trauma Surgery	United Kingdom	102	72 h	Yes
Chua et al. 2014	Early vs. late flap coverage for open tibial fractures	Journal of Orthopaedic Surgery	Singapore	89	72 h	Yes
Higgin et al. 2021	Outcomes following the delayed management of open tibial fractures	Injury	United Kingdom	114	168 h	No
Olesen et al. 2015	A review of forty-five open tibial fractures covered with free flaps. Analysis of complications, microbiology and prognostic factors	International Orthopaedics	Denmark	46	168 h	No
Singh et al. 2018	Gustilo IIIB Open Tibial Fractures: An analysis of Infection and Nonunion Rates	Indian Journal of Orthopaedics	Singapore	103	72 h	Yes

### Overview

All 5 included studies were published between 2014 and 2023. Of those 5 studies, 3 defined early orthoplastic coverage as definitive osteosynthesis of the open tibial fracture followed by definitive flap within 72 h after hospital admission. The other 2 studies defined the cutoff for early treatment as coverage within 168 h after hospital admission. Three studies utilized a single stage “fix and flap” approach (Table 2). All the included studies compared rates of FRI in the early coverage versus the late coverage group. Aside from Singh et al. [28] all studies also compared the amputation rates following early versus late treatment. Non-Union rates between early and late treatment groups were investigated by Aljawadi et al. [24], Higgin et al. [26] and Olsen et al. [27]. Aljawadi et al. [24] and Chua et al. [25] also compared revision rates in early and late treatment groups (see Table 2).

### Primary outcomes

**Fig. 2:**
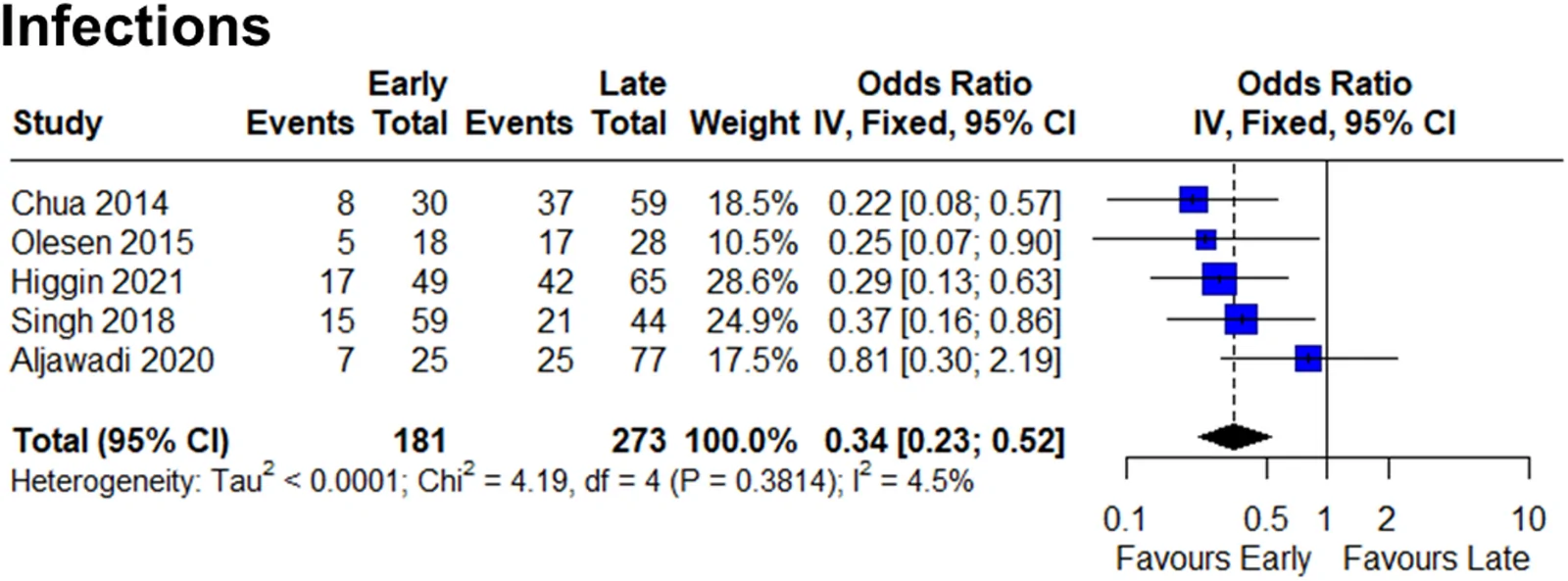
Forest plot comparing the odds for FRI between early and late orthoplastic coverage

Our primary outcome, the infection rate, was described in all 5 included studies. Out of 181 patients in the early treatment group 52 (28.7%) developed a FRI, out of the 273 patients in the late treatment group 142 (52%) developed a FRI as shown in Table 3a. Meta-analysis revealed significantly lower odds of infection in the early treatment group compared to the late treatment group (*OR*: 0.34; 95%CIs: 0.23 to 0.52; *p* < 0.001). The corresponding Forest plot is presented in Fig. 2. A comprehensive overview of all results from this analysis and from the subgroup analyses is presented in Table 3b.

**Table 3a:** Patient number and complication rates (primary and secondary outcomes) for each included study. “-“ = not reported

Total		Aljawadi 2020	Chua 2014	Higgin 2021	Olesen 2015	Singh 2018	Total
	*N* =	102	89	114	46	103	454
Early Orthoplastic Coverage	N =	25 (24.5%)	30 (33.7%)	49 (42.9%)	18 (39.1%)	59 (57.2%)	181 (39.8%)
Late Orthoplastic Coverage	N =	77 (75.5%)	59 (66.3%)	65 (57.1%)	28 (69.9%)	44 (42.8%)	273 (60.1%)
Complication Rate for early Orthoplastic Coverage	N =	25	30	49	18	59	181
	Infection	7 (28%)	8 (26.6%)	17 (34.6%)	5 (27.7%)	15 (25.4%)	52 (28.7%)
	Amputation	0	0	3 (6.1%)	3 (16.6%)	-	6 (3.3%)
	Non/Delayed Union	4 (16%)	-	4 (8.1%)	6 (33.3%)	-	11 (6.7%)
	Revision	8 (32%)	14 (46%)	-	-	-	22 (12.1%)
Complication Rate for late Orthoplastic Coverage	N =	77	59	65	28	44	273
	Infection	25 (32.4%)	37 (62.7%)	42 (64.6%)	17 (60.7%)	21 (47.7%)	142 (52%)
	Amputation	3 (3.8%)	4 (6.7%)	8 (12.3%)	1 (3.5%)	-	16 (5.8%)
	Non/Delayed Union	6 (8,8%)	-	13 (20%)	13 (46%)	-	28 (10.2%)
	Revision	12 (15%)	25 (42.3%)	-	-	-	37 (13.5%)

**Table 3b:** All results from meta-analyses (incl. test for heterogeneity)

	OR (Effect Size)	95% CI Lower	95% CI Upper	*p*.value	Regression Model	Z	I^2	Tau^2	Q	df	*p*.value Q-Stat
Infection	0.3444	0.2273	0.5223	> 0.0001	Fixed	-5.024	0.045	0.000	4.186	4	0.3814
Infection (72 h)	0.3943	0.2315	0.6722	0.0006	Fixed	-3.421	0.430	0.162	3.506	2	0.1732
Infection (168 h)	0.279	0.1434	0.5422	0.0002	Fixed	-3.766	0.000	0.000	0.042	1	0.8381
Infection (Fix & Flap)	0.4446	0.2508	0.7867	0.0054	Fixed	-2.782	0.148	0.030	2.348	2	0.3091
Infection (Staged)	0.2589	0.1413	0.4744	> 0.0001	Fixed	-4.373	0	0	0.220	1	0.6390
Amputations	0.5303	0.2169	1.2979	0.1649	Fixed	-1.389	0.340	0.662	4.547	3	0.2081
Non/Delayed Unions	0.742	0.2591	2.1254	0.5784	Random	-0.556	0.524	0.454	4.197	2	0.1226
Revision Operations	1.6368	0.8343	3.2114	0.1519	Fixed	1.433	0.163	0.047	1.194	1	0.2745

In the subgroup analysis of studies which set a cutoff time of 72 h (Chua et al., Singh et al. and Aljawadi et al.), the meta-analysis also revealed significantly lower odds of infection in the early treatment group (*OR*: 0.39; 95%CIs: 0.23 to 0.67; *p* < 0.001) as seen in Fig. 3a. In the two studies that defined a cutoff time of 168 h (Olesen et al. and Higgin et al.), the meta-analysis again showed significant lower odds of infection for the early treatment group (*OR*: 0.27; 95%CIs: 0.14 to 0.54; *p* < 0.001) as seen in Fig. 3b. Three of the included studies (Olesen et al., Singh et al. and Aljawadi et al.) utilized a single stage “fix and flap” approach. Meta-analysis of these three studies also revealed significantly lower odds of infection for the early treatment group (*OR*: 0.44; 95%CIs: 0.25 to 0.79; *p* = 0.005) as seen in Fig. 3c. On the other hand, meta-analysis of the two studies that utilized a staged approach also showed significantly lower odds of infection in the early treatment group (*OR*: 0.26; 95%CIs: 0.14 to 0.47; *p* < 0.001) (see also Table 3b).

**Fig. 3 A:**
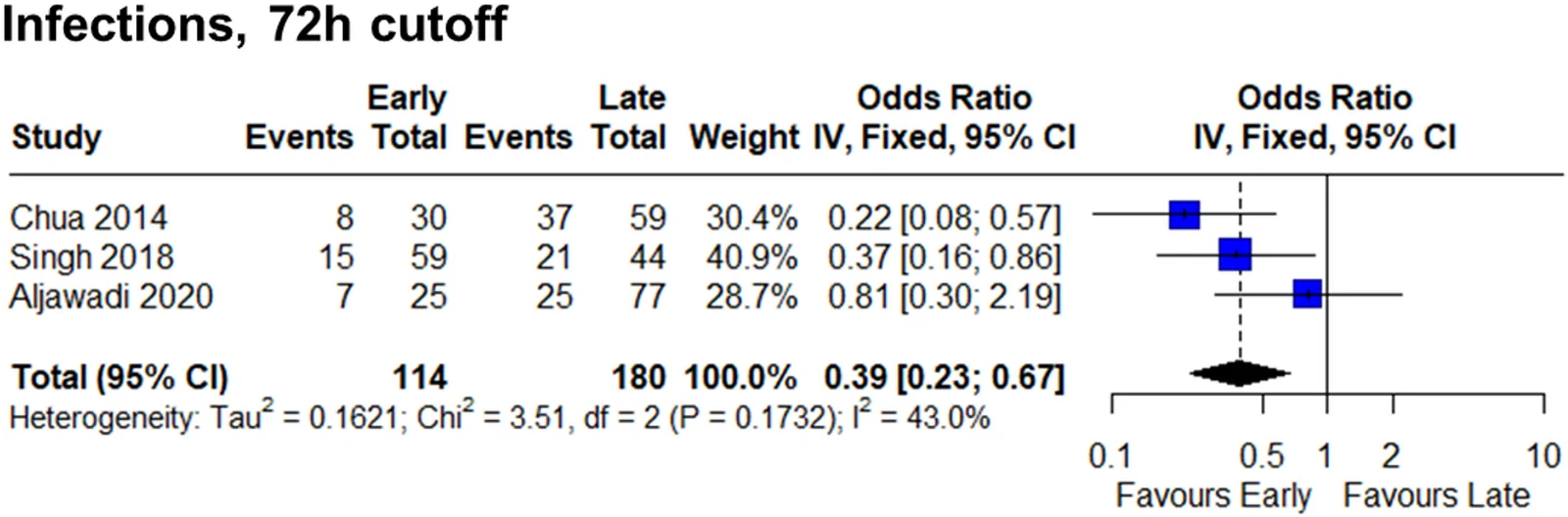
Forest plot comparing the odds for FRI between early and late orthoplastic coverage in studies with a 72 h cutoff

**Fig. 3B:**
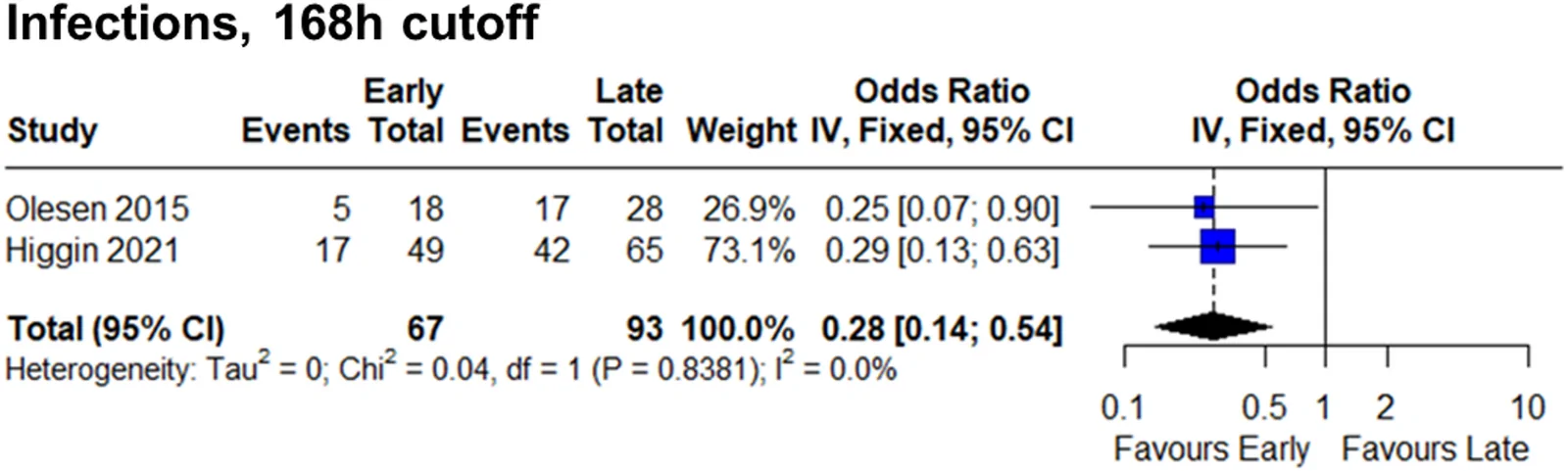
Forest plot comparing the odds for FRI between early and late orthoplastic coverage in studies with a 168 h cutoff

**Fig. 3 C:**
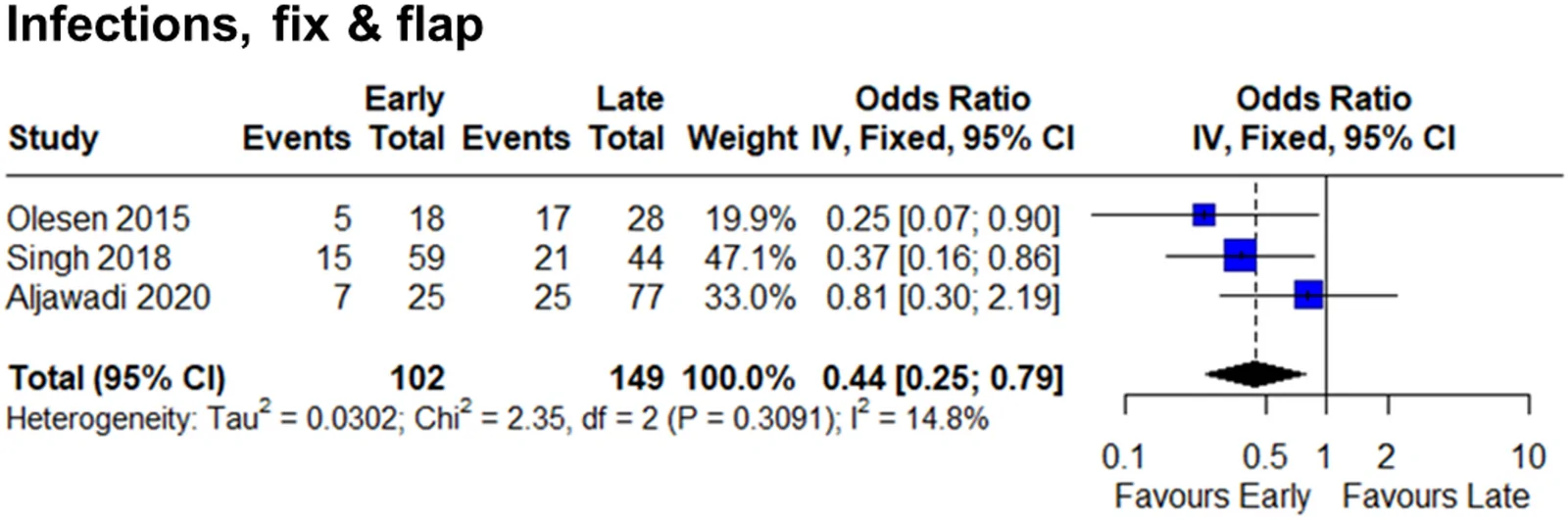
Forest plot comparing the odds for FRI between early and late orthoplastic coverage in studies that in which a single-stage “fix and flap” approach was utilized

### Secondary outcomes

For the three studies, which reported data on non-unions and delayed unions (Higgin et al., Olesen et al., and Aljawadi et al.), the meta-analysis revealed a non-significant trend towards a lower non/ delayed union rate (*OR*: 0.74; 95%CIs: 0.26 to 2.13; *p* = 0.578) as seen in Fig. 4a. Due to the moderate heterogeneity between the studies for this outcome (I^2^ = 0.52), a random effects model was chosen for only this analysis (See Table 3b). For the four studies that compared the amputation rates in the early versus the late treatment group (Chua et al., Higgin et al., Aljawadi et al., and Olesen et al.) the meta-analysis also revealed a non-significant trend favoring early orthoplastic coverage (*OR*: 0.53; 95%CIs: 0.22 to 1.30; *p* = 0.165) as seen in Fig. 4b. Our last secondary outcome, revision operations, was only reported by two of the included studies (Chua et al. and Aljawadi et al.). For the rate of revision operations, meta-analysis revealed a non-significant trend towards late operation (*OR*: 1.64; 95%CIs: 0.83 to 3.21; *p* = 0.152) as seen in Fig. 4c.

**Fig. 4a:**
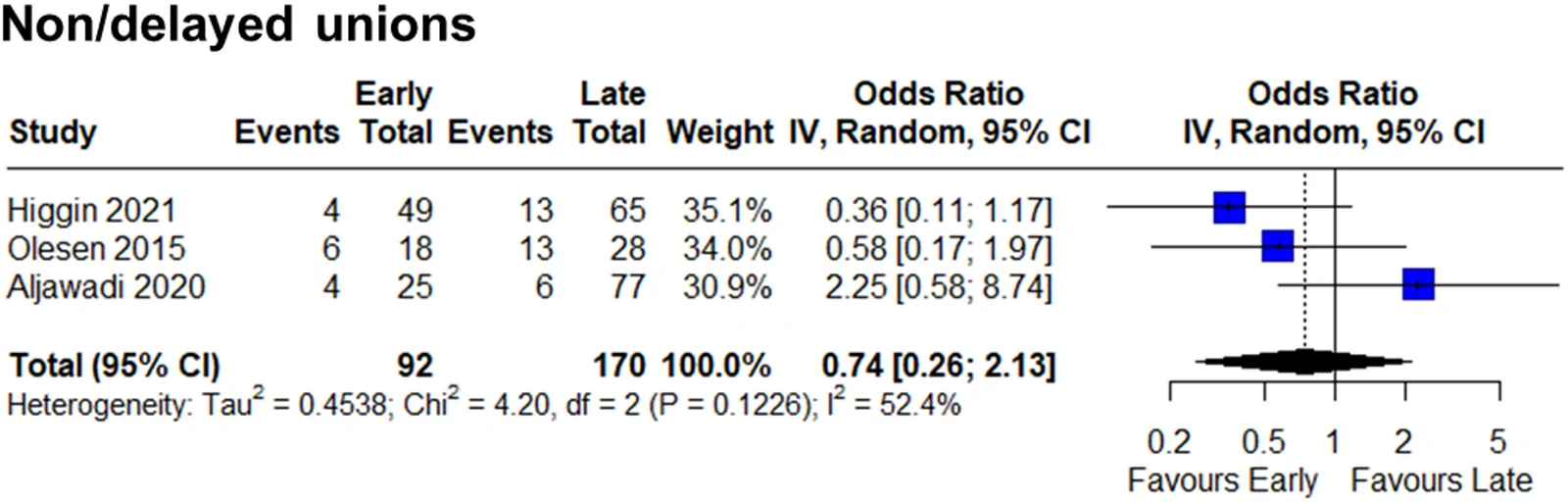
Forest plot comparing the odds for non/delayed unions between early and late orthoplastic coverage

**Fig. 4b:**
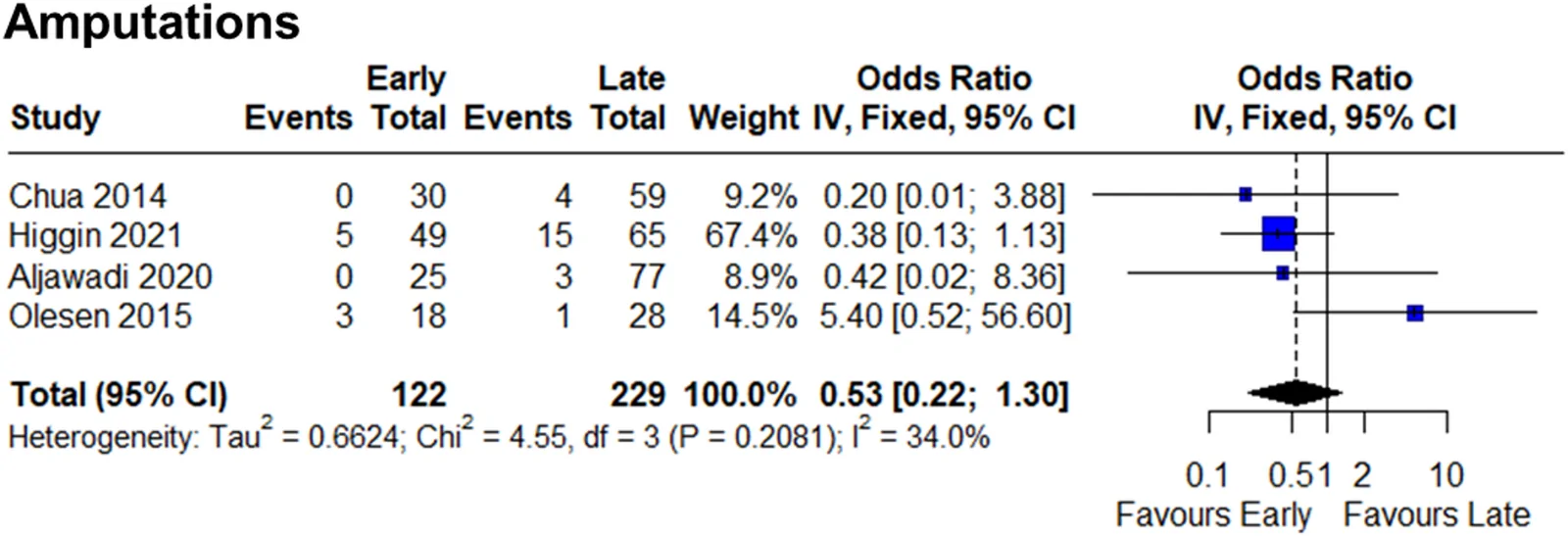
Forest plot comparing the odds for amputation between early and late orthoplastic coverage

**Fig. 4c:**
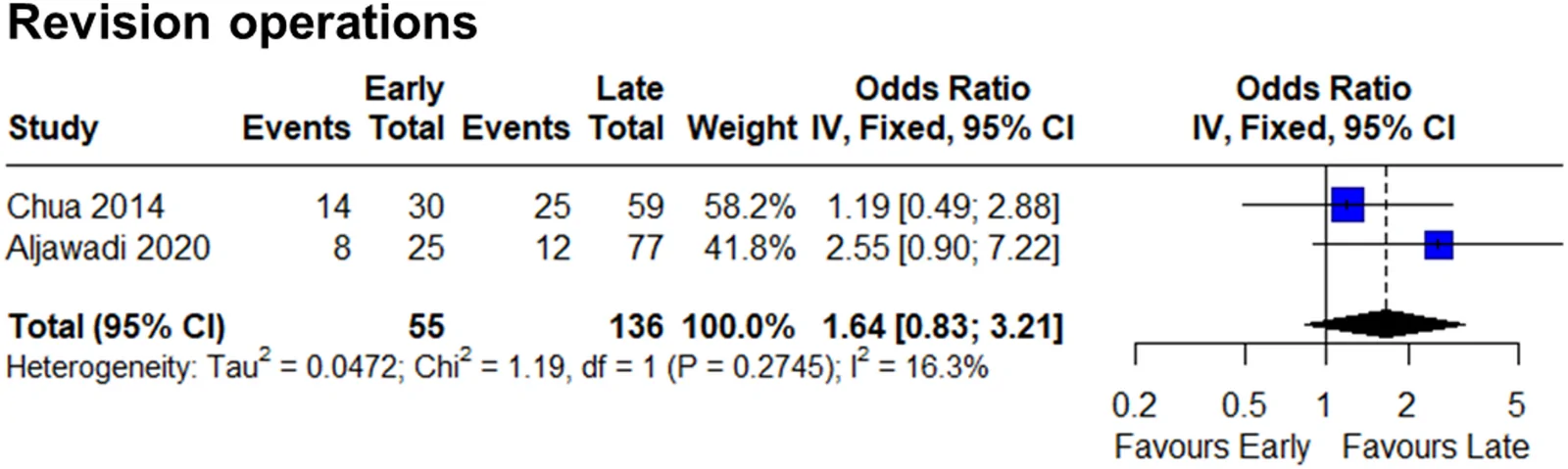
Forest plot comparing the odds for revision operations between early and late orthoplastic coverage

## Discussion

Gustilo-Anderson type III B/C fractures are characterized by extensive soft tissue injury, bone loss, severe contamination or vascular injury. They pose a significant challenge in terms of treatment and management, due to the high complication rates, especially regarding FRI [4, 5, 17, 29]. The socioeconomic impact of these fractures is substantial and prolonged hospitalization, revision operations, and prolonged rehabilitation results in personal and economic challenges for affected individuals [30, 31]. Regarding the treatment of those challenging injuries, our metanalysis has shown the following main results:

1. Early orthoplastic coverage was superior to late orthoplastic coverage across all included studies, especially regarding the odds of infection, which were significantly reduced.
2. This reduction in FRI remains significantly lower in the early treatment groups, regardless of whether the 72–168 h cutoff was chosen, or whether a staged protocol or a “fix and flap” strategy was employed.
3. We did not find a significant improvement regarding our secondary outcomes, however, early orthoplastic coverage shows a trend towards reduced amputation and non/delayed union rates.

Our first main result, the significantly reduced odds of infection in the early treatment group is very much in line with the published literature. In 2000 Gopal et al. and in 1999 Hertel et al. collectively highlighted that earlier definitive treatment of Gustilo-Anderson type III B and C fractures is associated with reduced infection rates, reinforcing the importance of prompt surgical management in these severe injuries [1, 32]. There are also more recent studies that have reinforced the importance of early orthoplastic coverage in reducing infection rates for these types of injuries [33, 34].

The significance of early orthoplastic treatment is also strongly emphasized in the BOAST guidelines of the United Kingdom, which recommend that these injuries be definitively treated within the first 72 h of hospitalization [7, 12]. The more recent SPARTA trial, one of the biggest trials up to date on this topic, further supports these guidelines in advocating for early definitive fixation [35]. Although the trial did not stratify between early and late definitive fixation and was therefore not included in our meta-analysis, it analyzed deep infection rates based on the time interval between definitive osseous fixation and flap coverage using a regression analysis. The findings demonstrated that delays beyond two days after osseous fixation were associated with a higher risk of FRI. Based on the results of our meta-analysis, we were unable to identify a universally applicable cutoff for the optimal timepoint of coverage. Our data clearly favoured earlier over delayed definitive treatment, but does not support a single rigid time threshold. The variability in the definitions of early vs. late definitive treatment mirrors differences in health system logistics. Studies that use a 72 h threshold often originate out of centres with co-located orthoplastic services with established procedures to accomplish definitive treatment within the 72 h threshold. The 168 h threshold on the other hand is a more realistic threshold for health systems in which achieving definitive treatment within 72 h is not consistently feasible, with transfer delays, polytrauma stabilization and limited immediate access to microsurgical teams, and a one week threshold, 168 h, provides a clinically meaningful distinction between timely and delayed definitive treatment. Therefore, the principle of ‘earlier is better’ must be judged in a real-world trauma care context, where factors such as the need for hemodynamic stability, polytrauma stabilization and inter-hospital transfer delays often limit the feasibility of very early definitive treatment.

Even though the literature seems to agree on the importance of early definitive orthoplastic coverage for Gustilo-Anderson type III B/C fractures, the specific requirements are yet to be investigated in more detail. Boriani et al., Fernandez et al. and Naique et al. promote a combined orthoplastic (“fix and flap”) approach as essential for optimal treatment [13, 36, 37]. This, however, can only be facilitated by a resource-heavy multidisciplinary approach, often unavailable in rural regions without a major trauma center in close vicinity. To compound this problem, as mentioned above, patients that needed to be transferred to definitive care trauma centers experience longer delays before definitive fixation, leading to less favorable functional outcomes as demonstrated in the study by Joost et al. [38]. The results from this meta-analysis, however, are insufficient to promote either a “fix and flap” or a staged approach.

Regarding our secondary outcomes (non-union, amputation, revision rates), the results from our analysis could not show a significant benefit, but they indicate a trend toward better secondary outcomes with earlier definitive treatment. A study by Tampe et al. demonstrated that patients who received definitive treatment within 72 h had significantly better clinical outcomes, including lower rates of deep infection, fewer revision surgeries, and improved fracture union rates [39]. Other studies, such as Aljawadi et al. [24] and Clegg et al. [40] support this finding.

### Strength & limitations

While the results of our meta-analysis are clear, there are several important limitations, which need to be discussed: First and foremost is the rather low level of evidence of the studies included: All included studies were relatively small, retrospective, non-randomized trials. The retrospective nature of these studies introduces potential biases, including selection bias and confounding factors, which may influence the validity of our findings. While there are few (prospective) studies with a larger patient population, these did not meet the inclusion criteria of comparing early versus late treatment with clearly defined outcome measures for both groups. Another limitation is that across the included papers there was a lack of standardized timing for flap coverage and this can lead to a significant variability in the interpretation of the outcomes and may affect the accuracy of our results. Furthermore, the number of included studies is small and as a result, the statistical power for detecting significant differences between the early and late groups was limited. From these limitations, it can be concluded that there is lack of higher-level evidence and that prospective (multicentric) studies with large patient populations are clearly needed [41].

Despite these limitations, our study has notable strengths. We identified five relatively homogenous studies that met our strict selection criteria, ensuring a rigorous and focused analysis. To our knowledge, this is the first meta-analysis to investigate this issue, thereby strengthening the level of evidence regarding this important topic. Additionally, these studies originated from three different continents, contributing to the international applicability of our meta-analysis.

## Conclusion

The results from our meta-analysis indicate that earlier definitive coverage of these complex injuries seems to have improved outcomes compared to later coverage. Given the strong association between early treatment and lower infection rates, we advocate for standardized treatment protocols to ensure timely, effective care. While our findings align with existing literature, the question of optimal timing for definitive treatment, particularly regarding secondary outcomes such as non-union, amputation, and revision rates, remains partially unanswered. To establish more definitive recommendations, higher-level evidence, including prospective and randomized controlled trials, are needed.

## Supplementary Information

Below is the link to the electronic supplementary material.

Supplementary Material 1

## Data Availability

No datasets were generated or analysed during the current study.

## Abbreviations

95%CIs: 95% Confidence Intervals
FRI: Fracture-Related Infection
OR: Odds Ratio
