# Supplementary material for: Early orthoplastic coverage reduces fracture-related infections in Gustilo III B/C tibial fractures – a systematic review and meta-analysis
Source: Eur J Trauma Emerg Surg. 2026 Apr 24;52(1):146. doi: 10.1007/s00068-026-03189-2 (PMC13731475; doi:10.1007/s00068-026-03189-2)
Supplement: Supplementary file 1 [file 68_2026_3189_MOESM1_ESM.docx]

**Supplemental material for** **”Timing Matters: Early Orthoplastic Coverage reduces Fracture-related Infections in Gustilo III B/C Tibial Fractures – A Systematic Review and Meta-Analysis”**

**Table S1:** Risk of Bias assessment according to the Newcastle-Ottawa Scale [21].

|  | Selection (Maximum 4 Stars) | Comparability (Maximum 2 Stars) | Outcome (Maximum 3 Stars) | Rating |
| --- | --- | --- | --- | --- |
| **Aljawadi** | **🟑🟑🟑** | **🟑** | **🟑🟑🟑** | Low Risk of Bias |
| **Chuah** | **🟑🟑🟑** |  | **🟑🟑** | Moderate Risk of Bias |
| **Higgin** | **🟑🟑🟑** | **🟑** | **🟑🟑🟑** | Low Risk of Bias |
| **Olesen** | **🟑🟑🟑** | **🟑** | **🟑🟑🟑** | Low Risk of Bias |
| **Singh** | **🟑🟑🟑** | **🟑** | **🟑🟑🟑** | Low Risk of Bias |

**Search Terms used:**

("Fractures, Open"[Mesh] AND "Tibial Fracture*"[Mesh]) OR

(tibia*[Title/Abstract] AND "open fract*" [Title/Abstract]) OR

"open tibia fract*"[Title/Abstract] OR

(tibia*[Title/Abstract] AND (IIIB [Title/Abstract] OR IIIC [Title/Abstract]) AND Gustilo[Title/Abstract])

"Surgical Flaps"[Mesh] OR

orthoplast*[Title/Abstract] OR

"ortho-plast*"[Title/Abstract] OR

cover*[Title/Abstract] OR reconstruct*[Title/Abstract] OR

"fix and flap"[Title/Abstract] OR "myocutaneous flap"[Title/Abstract] OR "muscle flap"[Title/Abstract] OR "fasciocutaneous flap"[Title/Abstract] OR "skin graft"[Title/Abstract] OR

timing[Title/Abstract] OR early[Title/Abstract] OR delayed[Title/Abstract] OR late[Title/Abstract] OR stage*[Title/Abstract]

**For Pubmed:**

Search: (("Fractures, Open"[Mesh] AND "Tibial Fracture*"[Mesh]) OR (tibia*[Title/Abstract] AND "open fract*" [Title/Abstract]) OR "open tibia fract*"[Title/Abstract] OR (tibia*[Title/Abstract] AND (IIIB [Title/Abstract] OR IIIC [Title/Abstract]) AND Gustilo[Title/Abstract])) AND ("Surgical Flaps"[Mesh] OR orthoplast*[Title/Abstract] OR "ortho-plast*"[Title/Abstract] OR cover*[Title/Abstract] OR reconstruct*[Title/Abstract] OR "fix and flap"[Title/Abstract] OR "myocutaneous flap"[Title/Abstract] OR "muscle flap"[Title/Abstract] OR "fasciocutaneous flap"[Title/Abstract] OR "skin graft"[Title/Abstract] OR timing[Title/Abstract] OR early[Title/Abstract] OR delayed[Title/Abstract] OR late[Title/Abstract] OR stage*[Title/Abstract]) Sort by: Most Recent

**For EMBASE:**

'open fracture'/exp AND 'tibia fracture'/exp OR

(tibia* AND "open fract*") OR

"open tibia fract*" OR

(tibia* AND (IIIB OR IIIC ) AND Gustilo)

'surgical flaps'/exp OR

orthoplast* OR

"ortho-plast*" OR

cover* OR reconstruct* OR

"fix and flap" OR "myocutaneous flap" OR "muscle flap" OR "fasciocutaneous flap" OR "skin graft" OR

timing OR early OR delayed OR late OR stage*
